# Overcoming the Epistemic Injustices Experienced by Older Adults in Health Innovations: How and What Can This Lead to?

**DOI:** 10.1093/geroni/igaf122.776

**Published:** 2025-12-31

**Authors:** Marie-Michele Lord, Valérie Poulin, Marie-Josee Drolet

**Affiliations:** Université du Québec à Trois-Rivières, Trois-Rivières, Quebec, Canada; Université du Québec à Trois-Rivières, Trois-Rivières, Quebec, Canada; Université du Québec à Trois-Rivières, Trois-Rivieres, Quebec, Canada

## Abstract

Epistemic injustices experienced by older adults refer to the marginalization of their knowledge, experiences, and perspectives, which are often ignored or devalued in social, cultural, and political discourses and decisions. This leads to limited inclusion of their perspectives when health innovations concerning them are developed, including those designed to support their social participation. This presentation aims to discuss a research project conducted with the goal of: 1) identifying factors associated with epistemic injustices when including the knowledge of older adults in the development of health innovations, and 2) implementing actions to counter these factors to co-create, with various societal actors, including older adults and their loved ones, a health innovation that places them at the center of medical and support interactions concerning them. This project was carried out using a living lab method, which is a participatory and collaborative research approach where participants (n = 40), from local communities, were actively involved in co-constructing knowledge and practical solutions. The results highlighted actors contributing to epistemic injustices were identified, including organizational disharmony and internalized ageism. The principles and values of a philosophical research community, and steps proposed by the Occupational Justice Collaborative Framework were used to address these factors. Multiple iterative working meetings with a co-development committee and an advisory committee led to the development of an innovative technological tool that enhances the agency and strengthens older adults’ social participation by fostering greater inclusion and engagement in decision-making processes that affect their lives.

